# CCAR2/DBC1 is required for Chk2-dependent KAP1 phosphorylation and repair of DNA damage

**DOI:** 10.18632/oncotarget.4417

**Published:** 2015-06-10

**Authors:** Martina Magni, Vincenzo Ruscica, Michela Restelli, Enrico Fontanella, Giacomo Buscemi, Laura Zannini

**Affiliations:** ^1^ Department of Experimental Oncology, Fondazione IRCCS Istituto Nazionale dei Tumori, Milan, Italy; ^2^ Department of Biosciences, University of Milan, Milan, Italy; ^3^ Current address: Max Planck Institute for Developmental Biology, Tubingen, Germany

**Keywords:** DNA damage, DNA repair, phosphorylation, chromatin relaxation

## Abstract

Cell cycle and apoptosis regulator 2 (CCAR2, formerly known as DBC1) is a nuclear protein largely involved in DNA damage response, apoptosis, metabolism, chromatin structure and transcription regulation. Upon DNA lesions, CCAR2 is phosphorylated by the apical kinases ATM/ATR and this phosphorylation enhances CCAR2 binding to SIRT1, leading to SIRT1 inhibition, p53 acetylation and p53-dependent apoptosis. Recently, we found that also the checkpoint kinase Chk2 and the proteasome activator REGγ are required for efficient CCAR2-mediated inhibition of SIRT1 and induction of p53-dependent apoptosis.

Here, we report that CCAR2 is required for the repair of heterochromatic DNA lesions, as cells knock-out for CCAR2 retain, at late time-points after genotoxic treatment, abnormal levels of DNA damage-associated nuclear foci, whose timely resolution is reinstated by HP1β depletion. Conversely, repair of DNA damages in euchromatin are not affected by CCAR2 absence.

We also report that the impairment in heterochromatic DNA repair is caused by defective Chk2 activation, detectable in CCAR2 ablated cells, which finally impacts on the phosphorylation of the Chk2 substrate KAP1 that is required for the induction of heterochromatin relaxation and DNA repair.

These studies further extend and confirm the role of CCAR2 in the DNA damage response and DNA repair and illustrate a new mechanism of Chk2 activity regulation. Moreover, the involvement of CCAR2 in the repair of heterochromatic DNA breaks suggests a new role for this protein in the maintenance of chromosomal stability, which is necessary to prevent cancer formation.

## INTRODUCTION

Human CCAR2 (cell cycle and apoptosis regulator 2, also known as DBC1 or KIAA1967) is a nuclear protein involved in several biological processes, such as DNA damage response (DDR) and apoptosis, cellular metabolism, epigenetics, cell proliferation and tumorigenesis, nuclear receptor function, circadian cycle and mRNA splicing [1]. Upon DNA damage, the apical checkpoint kinases ATM and ATR phosphorylate CCAR2 on T454, enhancing its inhibitory binding to the histone deacetylase SIRT1, promoting p53 acetylation and p53-dependent apoptosis [2, 3]. Besides phosphorylation, other CCAR2 post-translational modifications like acetylation and sumoylation finely regulate SIRT1 activity [4-6]. Additionally, the checkpoint kinase Chk2 and the proteasome activator REGγ do also play a role in the regulation of SIRT1 by CCAR2, as we recently reported [7].

Chk2 is a downstream component of the DDR [8], activated in response to DSBs by ATM, which phosphorylates Chk2 on T68, triggering its dimerization, auto-phosphorylation and activation. Chk2 phosphorylates several substrates, among which p53, Brca1, Cdc25C, PML, TRF2, KAP1 and REGγ, amplifying the DDR signaling and promoting cell cycle delay, DNA repair or apoptosis [7, 8].

Besides SIRT1, CCAR2 inhibits the activity of the histone-modifying enzymes SUV39H1 and HDAC3 [9, 10], thus playing an important role in chromatin structure regulation.

Chromatin relaxation is a key event in DDR as it favors the recruitment of repair factors at damaged sites. It is now well established that DNA lesions located in the tightly packaged heterochromatin are repaired with a slower kinetics compared to those occurring in the less compact and transcriptionally active euchromatin [11, 12]. Many proteins are involved in the regulation of chromatin structure; among them, the transcriptional co-repressor KAP1 (KRAB domain-associated protein 1) recruits histone deacetylases and methyltransferases to promote the transcriptionally inactive state of chromatin [13, 14]. Moreover, KAP1, which is also known to associate with CCAR2 [15], is involved in the recruitment of the heterochromatin protein 1 family (HP1α, HP1β e HP1γ) that binds methylated histones, preserving their methylation and promoting gene silencing [14, 16]. However, upon DNA damage KAP1 is phosphorylated by ATM on S824 [17] and by Chk2 on S473 [18, 19] inducing chromatin relaxation and DNA repair in the heterochromatic regions of the genome. Of note, phosphorylation of S473 by Chk2 decreases the interaction between KAP1 and HP1 proteins and is necessary for HP1β mobilization, a key event for DNA repair in the heterochromatin [18-21].

Here we report that, in human cells, CCAR2 loss markedly impairs the repair of DNA lesions in heterochromatin as consequence of a reduced kinase activity of Chk2 towards KAP1.

## RESULTS

### CCAR2 is required for the repair of DNA lesions

To thoroughly investigate the role of CCAR2 in the repair of DNA breaks, we generated U2OS cells knock-out for CCAR2 (CCAR2−/−) using the CRISPR/Cas9 system [22]. For our studies, we initially selected a U2OS clone characterized by the insertion of a single nucleotide in both strands of CCAR2 gene (alignment is shown in Supplementary Figure 1A and sequence chromatogram in Supplementary Figure 1B), which caused a premature stop codon formation and complete loss of CCAR2 protein expression. The absence of CCAR2 was further confirmed by immunofluorescence analyses performed with two different anti-CCAR2 antibodies recognizing epitopes at the N-terminus (Supplementary Figure 1C, right) and C-terminus (Supplementary Figure 1C, left), and by western blot (Supplementary Figure 1D).

Next, we assessed in these cells the repair of DNA damages induced by etoposide treatment, a chemotherapeutic drug that inhibits topoisomerase II, finally inducing double strand breaks (DSBs), and that is known to strongly promote ATM/ATR-dependent phosphorylation of CCAR2 and apoptosis [2]. Although etoposide is known to induce DNA lesions mainly in S-G2 phases of the cell cycle, we found that, at the dose we used (20μM), etoposide can induce DSBs in all cells. Indeed immunofluorescence staining with the DSBs marker γH2AX demonstrated that all cells are damaged 1h after etoposide treatment, as previously reported [23, 24], and these lesions are partially repaired 24h later (Supplementary Figure 2).

**Figure 1:**
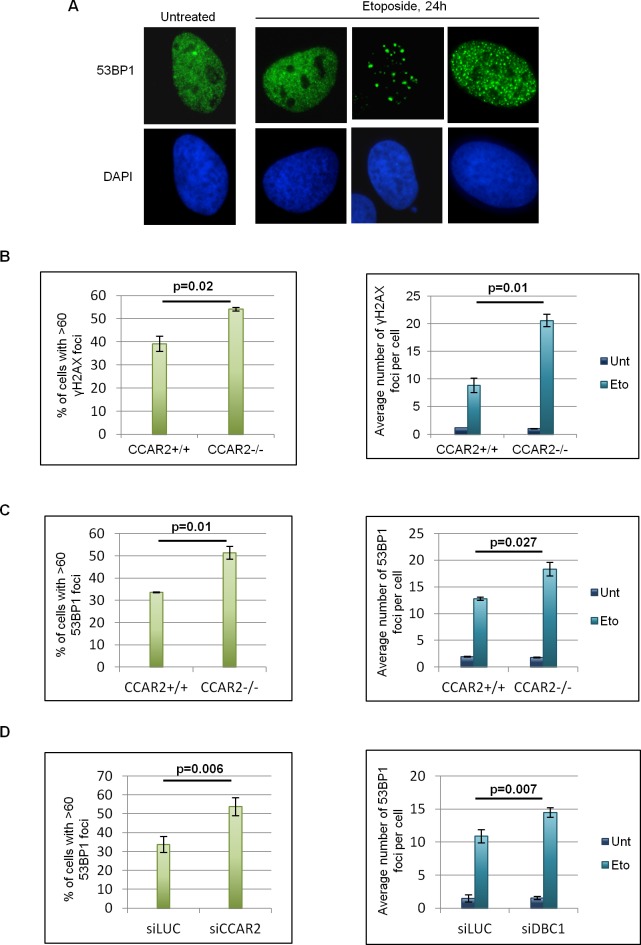
Cells negative for CCAR2 have defective DNA repair **A.** Examples of 53BP1 IF staining in U2OS cells before and 24h after etoposide exposure. **B.** Charts depicting the percentage of cells with >60 γH2AX foci in U2OS CCAR2+/+ and CCAR2−/− cells 24h after etoposide exposure (left) and the average number of γH2AX foci detected in CCAR2+/+ and CCAR2−/− cells with less than 60 foci before and 24h after etoposide treatment (right). **C.** Charts obtained as in B, but with 53BP1 staining **D.** Charts depicting the percentage of cells with >60 53BP1 foci in U2OS siLUC and siCCAR2 cells 24h after etoposide exposure (left) and the average number of 53BP1 foci detected in cells with less than 60 foci before and 24h after etoposide treatment (right). Results are the mean and standard deviation of at least 3 independent experiments. p values indicate statistically significant differences.

Repair of DNA breaks is bimodal, with those in euchromatin being repaired within few hours following damage and those in heterochromatin much later, necessitating chromatin relaxation for repair [11]. As CCAR2 appears involved in chromatin dynamics through its repression of the histone modifying enzymes SIRT1, SUV39H1, HDAC3 and interaction with KAP1 [2, 3, 9, 10, 15], we especially investigated the late repair of DNA lesions which critically depends on chromatin remodeling functionality. Specifically, we analyzed by immunofluorescence (IF) the formation and clearance of γH2AX and 53BP1 nuclear foci, two biomarkers of DSBs [25], in U2OS CCAR2+/+ and CCAR2−/− cells treated with etoposide for 1h, and then incubated in drug-free medium for 24h as previously reported [18]. Although no differences were found in the levels of DSBs at 1-3h after treatment, since 53BP1 foci and γH2AX levels were similar in CCAR2+/+ and CCAR2−/− cells (Supplementary Figure 3A and 3B), the 53BP1 and γH2AX staining, at 24h, revealed three subsets of nuclei exhibiting either large numbers of foci (>60), less than 60 foci, or no foci (Figure 1A, Supplementary Figure 2 and 3A). Notably, however, immunostaining of γH2AX (Figure 1B) and 53BP1 (Figure 1C) revealed that both the fraction of cells containing >60 foci and the overall number of foci in the remaining cells were markedly higher in CCAR2−/− than in CCAR2+/+ cells and similar results were also obtained by staining of 53BP1 in U2OS cells transfected with control or CCAR2 siRNA (Figure 1D and Supplementary Figure 3C), thus excluding a clone specific effect. In accordance with these data, the percentage of cells with repaired DNA lesions (less than 5 foci) is strongly reduced in CCAR2−/− compared to CCAR2+/+ cells, as evident from the chart showing foci number versus cells distribution (Supplementary Figure 3D).

Moreover, the role of CCAR2 in the repair of DSBs was further confirmed in time course analyses of 53BP1 foci in etoposide treated BJ-hTERT human fibroblast cells where CCAR2 gene was knocked-out by the CRISPR/Cas9 system (Supplementary Figure 3E). Analysis of a BJ-hTERT-CCAR2−/− clone revealed that this protein is required for efficient repair of DSBs, after genotoxic treatment and, thus, this CCAR2 function is not restricted to cancer cells.

To investigate if accumulation of cells with unrepaired DNA breaks in CCAR2 ablated cells could be due to alterations of cell cycle progression induced by CCAR2 absence, we performed FACS analyses [26] of U2OS CCAR2+/+ and CCAR2−/− cells, before and after damage, and found similar cell cycle profile in both cell lines (Supplementary Figure 4).

**Figure 2:**
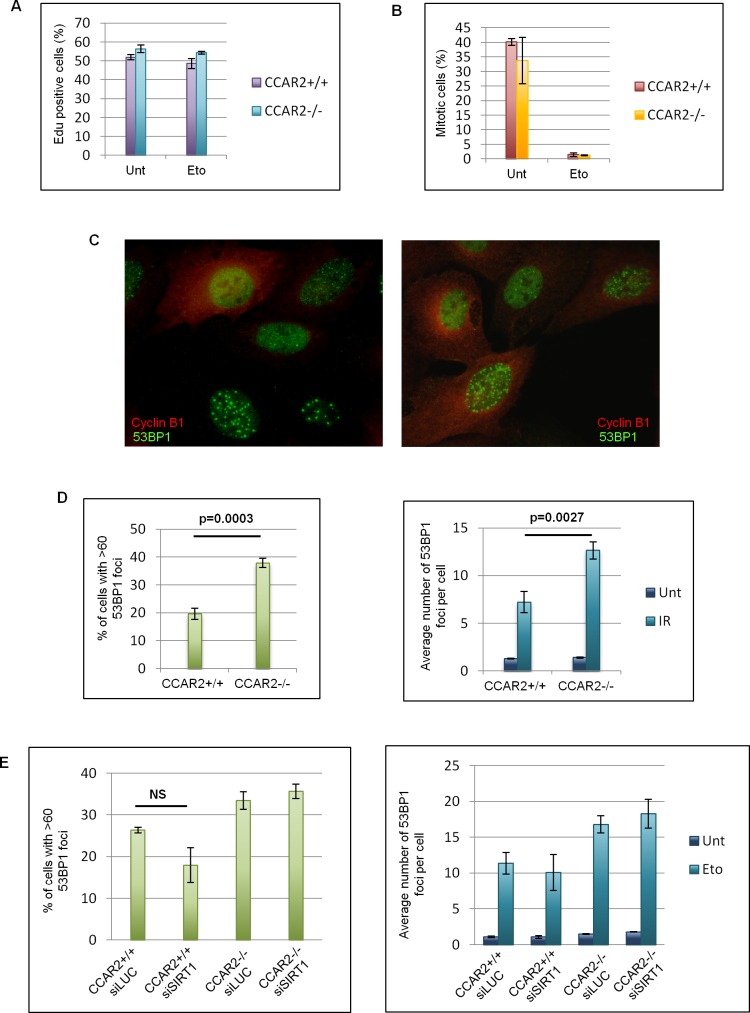
The DNA repair defect of CCAR2 negative cells is not cell cycle dependent **A.** U2OS CCAR2+/+ and CCAR2−/− cells were treated with etoposide for 1h and released in EdU containing medium for 4h. EdU positive cells were stained, enumerated and obtained data were reported in the chart. **B.** U2OS CCAR2+/+ and CCAR2−/− cells were treated with etoposide for 1h and released in nocodazole containing medium for 24h to trap mitotic cells. Obtained results were reported in the chart. No significant differences between CCAR2+/+ and CCAR2−/− cells were detected in S phase progression nor in G2/M transition. **C.** Examples of cyclin B1 and 53BP1 double immunostaining. **D.** Charts depicting the percentage of cells with >60 foci in U2OS CCAR2+/+ and CCAR2−/− cells 24h after IR exposure (left) and the average number of 53BP1 foci detected in CCAR2+/+ and CCAR2−/− cells with less than 60 foci before and 24h after IR treatment (right). **E.** Charts depicting the percentage of cells with more than 60 foci (left) and the average number of foci in the remaining cells (right) in CCAR2+/+ and CCAR2−/− cells silenced or not for SIRT1. NS, not significant difference (*p* = 0.1).

To deepen investigate this point, we studied S-phase progression and G2/M transition of CCAR2+/+ and CCAR2−/− cells. For this, cells treated with etoposide for 1h, were released respectively in EdU or nocodazole containing medium and then EdU positive cells (corresponding to S-phase progressing cells; Figure 2A) and phospho-Histone-H3 (Ser10) positive cells (corresponding to mitotic cells; Figure 2B) were enumerated [26]. As shown in the charts, no significant differences between CCAR2+/+ and CCAR2−/− cells were found, thus suggesting that the DNA repair defect observed in CCAR2 depleted cells is not due to defects in checkpoint activation. In addition, findings that cells with persistent DNA damage induced foci can be both cyclin B1 positive or negative (a marker of G2 cells, Figure 2C) further confirm that damaged cells are not in a specific cell cycle phase.

Finally, to verify if CCAR2 could be involved in the repair of DNA lesions caused by genotoxic agents different from etoposide and capable to induce DSBs in all cell cycle phases, we exposed CCAR2+/+ and CCAR2−/− cells to ionizing radiation (IR). Staining and enumeration of 53BP1 foci in these cells revealed that CCAR2 ablation prevents the correct repair of DSBs also in response to IR (Figure 2D).

Since SIRT1 is the main CCAR2 target in the DNA damage response, we verified whether this protein could have some role in CCAR2 mediated DNA repair. For this CCAR2+/+ and CCAR2−/− cells were transfected with control or SIRT1 siRNAs and 53BP1 foci were analysed in response to etoposide treatment. However no significant differences were found between control and SIRT1 depleted cells (Figure 2E and Supplementary Figure 5A).

Altogether these results suggest that CCAR2 is required for the repair of DSBs in both normal and cancer cells and that this CCAR2 function is cell cycle and SIRT1 independent.

### CCAR2 is involved in heterochromatic DNA repair

**Figure 3:**
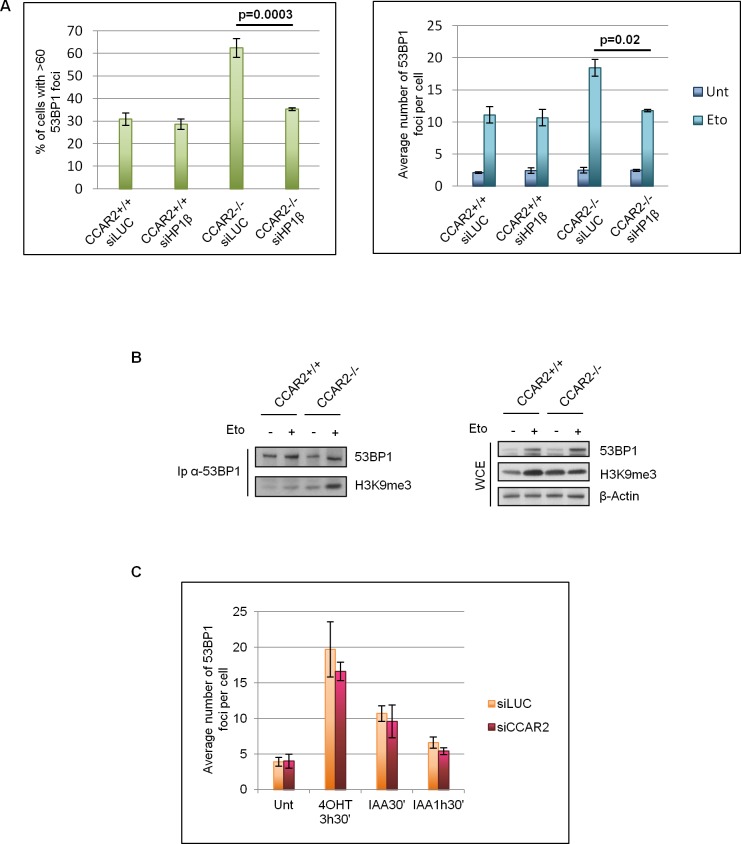
CCAR2 is involved in heterochromatic DNA repair **A.** Charts depicting the percentage of cells with more than 60 foci (left) and the average number of foci in the remaining cells (right) in CCAR2+/+ and CCAR2−/− cells silenced or not for HP1β. **B.** Coimmunoprecipitation analysis of 53BP1 and H3K9me3 in U2OS-CCAR2+/+ and CCAR2−/− cells before and after etoposide treatment; right panel: input. **C.** Analysis of 53BP1 foci in AID-DIvA cells transfected with control or CCAR2 siRNA in response to 4-hydroxytamoxifen (4-OHT) and auxin (IAA) treatments for the indicated time points.

Since CCAR2 seems to be involved in chromatin remodelling and these events are particularly important for the repair of heterochromatic DNA lesions which requires chromatin relaxation, we investigated if the foci retained in CCAR2 negative and depleted cells correspond to heterochromatic DSBs. It was previously demonstrated that depletion of proteins of the HP1 family can alleviate the defects in the repair of heterochromatic DSBs [19]; particularly HP1β mobilization seems to be a key event for the reorganization of chromatin structure and repair of DNA breaks in the heterochromatin [18, 19]. Thus, to verify if CCAR2 depletion affects the repair of DNA breaks in heterochromatin, we depleted HP1β by siRNA in U2OS CCAR2+/+ and CCAR2−/− cells and enumerated 53BP1 foci 24h after etoposide treatment. Significantly, HP1β ablation rescued the DNA repair defect of etoposide-treated CCAR2−/− cells (Figure 3A and Supplementary Figure 5B and 5C). Then, to further confirm that the late 53BP1 foci detectable in CCAR2 null cells correspond to heterochromatic DNA lesions, we analysed the levels of the heterochromatic marker H3K9me3 associated with 53BP1 positive polynucleosomes. As shown in Figure 3B, the amount of 53BP1 co-precipitating with H3K9me3 strongly increased after damage in U2OS CCAR2−/− compared to CCAR2+/+ cells.

To further confirm that the DNA repair defect detectable in CCAR2 ablated cells could be ascribed to impairment in the repair of heterochromatic DSBs, we took advantage of U2OS AID-DIvA cells [27]. These cells are characterized by the inducible nuclear translocation of the AID-AsiSI enzyme, which is able to cut the genome at known sites, but only in the euchromatic regions, and that can be turned off by auxin addition. AID-DIvA cells were transfected with control or CCAR2 siRNA and the induction and repair of DNA lesions followed by 53BP1 staining. Foci were enumerated and the data reported in the chart clearly demonstrate that there are no significant differences between control and CCAR2 depleted cells and thus CCAR2 is not involved in the repair of euchromatic DNA breaks (Figure 3C and Supplementary Figure 6).

Collectively, these data indicate that CCAR2 is required for the repair of DSBs localized in the heterochromatic regions of the genome.

### CCAR2 regulates Chk2 activity towards KAP1

As CCAR2 is known to interact with ATM [3], a kinase also required for heterochromatic DNA repair [17, 20], we first checked the impact of CCAR2 overexpression on ATM activity. For this, etoposide-treated U2OS-CCAR2+/+ and CCAR2−/− cells were harvested 6h later and analysed for the phosphorylation of the ATM targets Chk2 and KAP1. However, neither the phosphorylation of Chk2-T68 nor that of KAP1-S824 was influenced by CCAR2 overexpression, a finding consistent with the autophosphorylation of ATM-S1981, which is unaffected by CCAR2 (Supplementary Figure 7).

**Figure 4:**
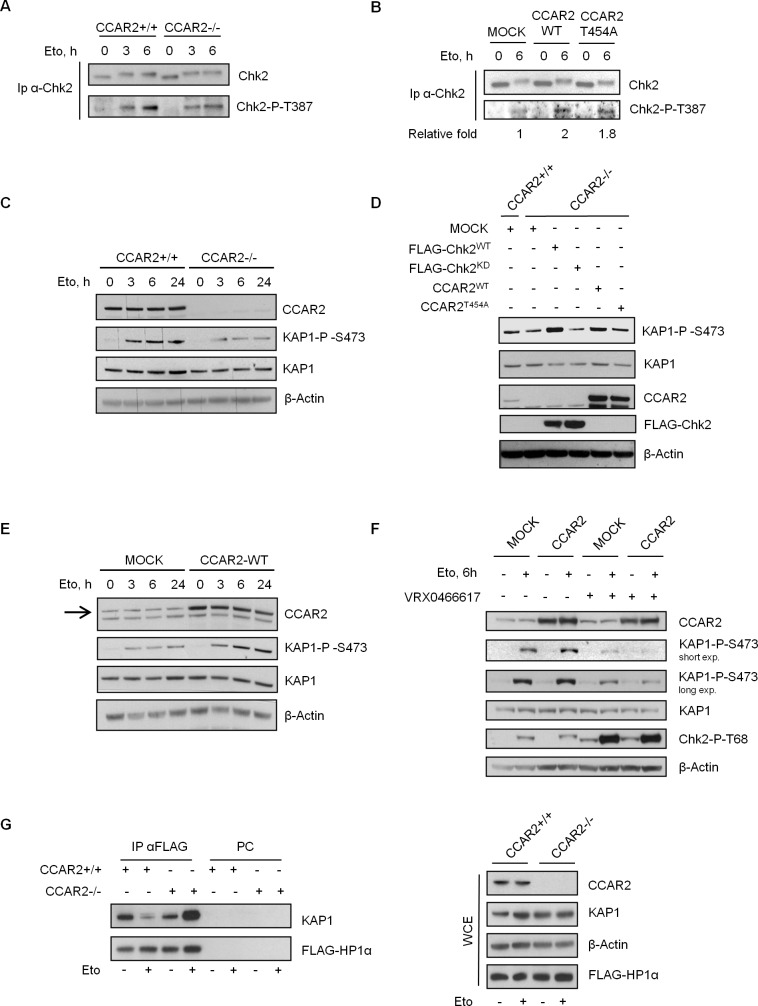
CCAR2 is required for Chk2 activation Chk2 immunoprecipitates from CCAR2+/+ and CCAR2−/− U2OS cells **A.**, or from CCAR2^WT^ and CCAR2^T454A^ overexpressing cells **B.** were analyzed for Chk2-T387 autophosphorylation before and after etoposide exposure. **C.** Time course analysis of KAP1-S473 phosphorylation in CCAR2+/+ and CCAR2−/− cells exposed to etoposide. **D.** KAP1-S473 phosphorylation in CCAR2−/− cells transfected with WT or KD Chk2 or CCAR2^WT^ and CCAR2^T454A^ encoding vectors and treated with etoposide for 6h. **E.** Time course analysis of KAP1-S473 phosphorylation in etoposide treated CCAR2-overexpressing cells. Arrow indicates CCAR2 specific band. **F.** Phosphorylation of KAP1-S473 in CCAR2-overexpressing cells treated or not with etoposide and VRX0466617. Accumulation of Chk2-pT68 demonstrates VRX0466617 efficacy [28]. **G.** Association of KAP1 and HP1α in CCAR2+/+ and CCAR2−/− cells before and 24h after etoposide treatment for 1h (left). PC: pre-cleared negative control. Right panel: input.

We previously reported that Chk2, like ATM, interacts with CCAR2 [7]. As Chk2 phosphorylation of KAP1-S473 induces the mobilization of HP1 from heterochromatin and promotes chromatin relaxation [18, 19], we investigated whether the defects in heterochromatic DNA repair in CCAR2-deficient cells was somewhat linked to Chk2. To this aim, we initially analyzed Chk2 autophosphorylation on T387 in CCAR2+/+ and CCAR2−/− U2OS cells, and, quite surprisingly, found a reduced Chk2-pT387 signal in the latter cells at 6h of etoposide treatment (Figure 4A). Conversely, the expression of CCAR2^WT^ led to a twofold increase in Chk2-pT387 signal, and almost a similar increase was seen by the expression of CCAR2^T454A^ phosphomutant [2] (Figure 4B), clearly indicating that, in human cells, CCAR2 positively regulates Chk2 autophosphorylation.

Next, we analyzed the time-dependent KAP1-S473 phosphorylation after etoposide treatment. As shown in Figure 4C, the absence of CCAR2 reduced the phosphorylation of KAP1-S473 and similar results were obtained also in BJ-hTERT-CCAR2−/− cells (Supplementary Figure 8) and after CCAR2 silencing and in response to IR (data not shown), further confirming that CCAR2 regulates Chk2 activity towards KAP1. To verify the specificity of these events, we transfected U2OS-CCAR2−/− cells with vectors encoding CCAR2^WT^, CCAR2^T454A^, FLAG-Chk2^WT^ and FLAG-Chk2^KD^ (kinase dead, a catalytically inactive mutant) and, after etoposide treatment, we found that expression of CCAR2 (both the wild type and, to a lesser extent, the T454A mutant) and wild type Chk2 restores KAP1-S473 phosphorylation, but not the expression of Chk2^KD^ (Figure 4D), indicating that the defective KAP1 phosphorylation on S473 is caused by CCAR2 deficiency which in turn affects Chk2 activity.

Moreover, in accordance with these data we found that the overexpression of CCAR2 in U2OS cells resulted in increased KAP1-S473 phosphorylation (Figure 4E) at all time points after etoposide treatment, and this event was Chk2-dependent as it was abrogated by pre-treatment of cells with the Chk2-specific inhibitor VRX0466617 [28] (Figure 4F).

Since KAP1 phosphorylation on S473 regulates its interaction with HP1 family members [18], we investigated this association in relation to CCAR2 expression. For this, CCAR2+/+ and CCAR2−/− U2OS cells were transfected with FLAG-HP1α and analyzed by immunoprecipitation for KAP1-HP1α interaction before and after etoposide treatment. While in U2OS-CCAR2+/+ cells etoposide treatment led to a reduced KAP1-HP1α binding, in accordance with previous reports [18], this treatment markedly enhanced the KAP1-HP1α association in CCAR2−/− cells (Figure 4G), an increase which could be explained by the defective KAP1 phosphorylation that prevents its dissociation from HP1α.

These findings further confirm the importance of CCAR2 in chromatin dynamics following DNA damage and that the DNA repair defect of CCAR2 knock-out cells can be ascribed to a defective Chk2 functionality on KAP1. Given however that CCAR2 silencing does not affect the proapoptotic function of Chk2 [7], a finding further substantiated here in CCAR2−/− cells (Supplementary Figure 9A), nor the phosphorylation of p53 on S20 [8] (Supplementary Figure 9B), it is likely that CCAR2 might differentially regulate the activity of Chk2 towards distinct targets.

### The CCAR2-dependent failure of DNA repair is caused by defective Chk2 activity

**Figure 5:**
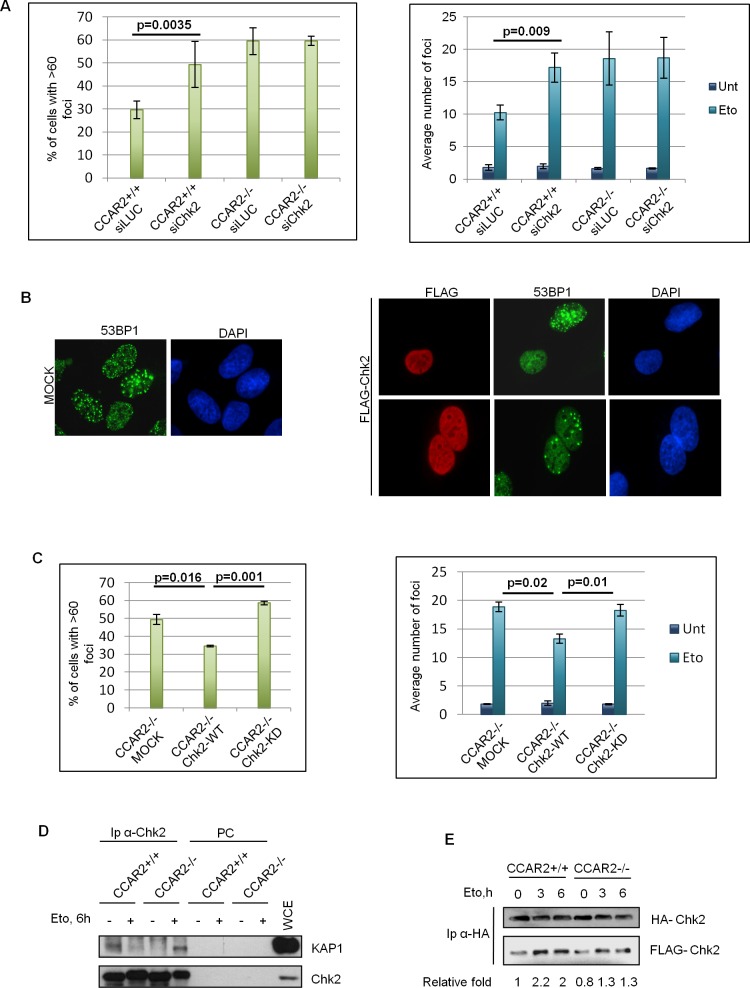
Chk2 transfection restores DNA repair in CCAR2 negative cells **A.** Chk2 was silenced in U2OS CCAR2+/+ and CCAR2−/− cells and the percentage of cells with >60 foci (left) and the average number of foci in the remaining cells (right) were evaluated after etoposide treatment. **B.** Example of 53BP1 staining in etoposide treated CCAR2−/− cells transfected with mock or Chk2 encoding vectors. **C.** Percentage of cells with more than 60 foci (left) and average number of foci in the remaining cells (right) in CCAR2−/− cells transfected with mock, Chk2^WT^ or Chk2^KD^ vectors 24h upon etoposide exposure. Charts represent the mean and standard deviation of three independent experiments, with significant *p*-values indicated. **D.** Co-IP between Chk2 and KAP1 before and after DNA damage in CCAR2+/+ and CCAR2−/− cells. PC: pre-cleared negative control. **E.** FLAG-Chk2 and HA-Chk2 encoding vectors were transfected in CCAR2+/+ and CCAR2−/− cells. Homodimerization was evaluated by analysis of FLAG-tagged Chk2 in HA-tagged Chk2 immunocomplexes. Relative fold indicates the densitometric quantification of FLAG-Chk2 co-immunoprecipitated with HA Chk2; data were normalized to CCAR2+/+ untreated sample.

We reasoned that if Chk2 is involved in CCAR2-dependent repair of heterochromatic DSBs, its depletion might predictably result in a similar repair defect as CCAR2 deficiency, and furthermore if Chk2 and CCAR2 work epistatically, then ablation of both proteins should not have an additive effect. To test this hypothesis U2OS CCAR2+/+ and CCAR2−/− cells were transfected with control or Chk2 siRNA and 53BP1 foci enumerated before and after etoposide treatment. As reported in the graphs (Figure 5A and Supplementary Figure 10), Chk2 knockdown, in CCAR2+/+ cells, induced a significant accumulation of cells with >60 foci and an increase in the average number of foci in the remaining cells, but this effect was not additive with that induced by CCAR2 ablation. Hence, Chk2 and CCAR2 appear to function in the same pathway to regulate the repair of heterochromatic DNA lesions.

To further verify that the DNA repair deficiency of CCAR2−/− cells is indeed due to defective Chk2 activity, 53BP1 foci were assessed 24h after etoposide treatment in U2OS-CCAR2−/− cells transfected with Chk2^WT^ or Chk2^KD^ vectors and, as shown in Figure 5B-5C, cells expressing Chk2^WT^ had fewer 53BP1 foci than mock or Chk2^KD^ expressing cells, indicating that increased Chk2 activity can rescue the repair defect caused by CCAR2 deficiency.

To get a more in-depth picture of the regulation of Chk2 activity by CCAR2, we analysed the association between Chk2 and KAP1 in CCAR2+/+ and CCAR2−/− cells. Interestingly, whereas in CCAR2-WT cells the binding between Chk2 and KAP1 decreased upon DNA damage, possibly because Chk2 releases its substrates after phosphorylation as previously reported [8, 29, 30], in CCAR2-KO cells this binding increased (Figure 5D), concordant with a defective Chk2 activity towards KAP1. Moreover, since Chk2 homodimerization is required for its autophosphorylation and activity, we determined whether the catalytic defect observed in CCAR2 deficient cells could be ascribed to impaired homodimerization. For this, CCAR2+/+ and CCAR2−/− cells were transfected with vectors encoding HA-Chk2 and FLAG-Chk2, ectopic proteins were immunoprecipitated with the anti-HA antibody and immunoblotted with anti-FLAG as previously reported [31]. As shown in Figure 5E, Chk2 homodimerization was induced in response to DNA damage, especially at 3h of treatment, in CCAR2-WT, but not in CCAR2-KO cells. Altogether, these results suggest that CCAR2 might affect Chk2 autophosphorylation and activity towards its substrates by regulating its homodimerization and interaction with target proteins.

**Figure 6:**
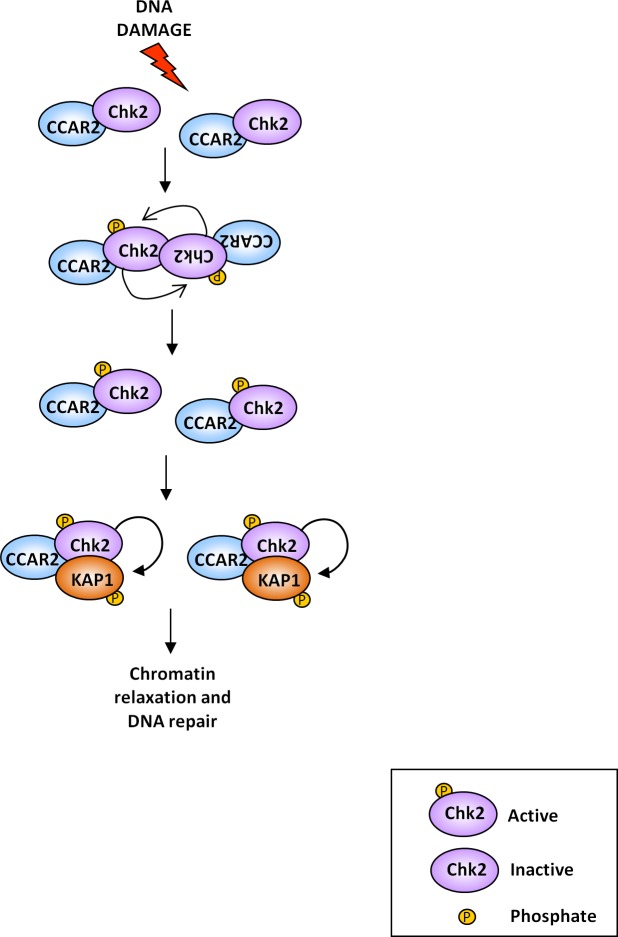
Graphical representation of the CCAR2 role in Chk2 activation and DNA repair In unstressed cells Chk2 kinase exists as inactive monomer. Upon DNA damage, CCAR2 contributes to Chk2 homodimerization and activation by autophosphorylation, which induces KAP1 phosphorylation on S473, thus increasing DSBs repair, possibly by induction of chromatin relaxation.

Collectively our data indicate that, in response to DNA damage, CCAR2 favours Chk2 dimerization and activation, leading to KAP1 phosphorylation, which is required for heterochromatic DNA repair by regulation of chromatin relaxation (Figure 6). These findings are consistent with the preferential repair of heterochromatic DNA breaks by homologous recombination [32], in which CCAR2 is involved [33], and suggest a new mechanism of Chk2 activity regulation.

## DISCUSSION

Here, we describe a novel function for CCAR2 implicated in the repair of DNA lesions in heterochromatic regions of the genome. Notably, these events are not mediated by CCAR2 dependent inhibition of SIRT1, the main CCAR2 target in the DNA damage response [34, 35], but, instead, they seem to be dependent on defective Chk2 activity regulation.

To evaluate DNA repair, we analyzed the formation and clearance of γH2AX and 53BP1 foci, two well-known markers of DSBs [25], in U2OS and BJ-hTERT human cells. Particularly, 24h after damage induction by both etoposide and IR, we observed the presence of cells with un-repaired DNA lesions and therefore a high number of γH2AX and 53BP1 positive foci. Thus this phenomenon is irrespective of the source of DSBs since etoposide mainly produces breaks during S and G2 phases of the cell cycle, whereas IR can damage cells in all cell cycle phases. These defects in DNA repair are present in highly cycling U2OS cells and slowly growing BJ-hTERT cells and do not derive from alterations of cell cycle progression since CCAR2 depletion does not affect cell cycle distribution of untreated cells nor checkpoint activation after damage. Moreover, staining with cyclin B1 (a marker of G2 phase cells) demonstrated that cells with a high number of foci are not all in the same phase of the cell cycle. Thus, we hypothesize that cells with a high amount of foci (>60), 24h after damaging treatment, are unable to repair DNA breaks and could be committed to death.

As previous reports suggest that CCAR2 could be implicated in the regulation of chromatin remodelling through its interaction with SIRT1, HDAC3, SUV39H1 and KAP1 [2, 3, 9, 10, 15], we hypothesized that CCAR2 could be necessary for the repair of those DNA breaks which require chromatin modification. It is now well established that DSBs which are repaired at late time points after DNA damage induction and necessitate chromatin relaxation, are those localized in the more compact heterochromatic regions of the genome [11, 12]. Thus, we investigated if the DNA repair deficiency detectable in CCAR2 negative cells could be ascribed to defective heterochromatic repair. Indeed, we found that depletion of HP1β, which induces chromatin relaxation [19], can abrogate the defect caused by CCAR2 absence. Moreover, in CCAR2−/− cells, retaining an elevated number of foci, 53BP1 strongly interacts with H3K9me3. Conversely, when we induced DNA damages in only euchromatic regions, using the AID-DIvA cells [27], no defects were detected in CCAR2 depleted cells, further confirming that CCAR2 is involved in the repair of heterochromatic DNA lesions.

These findings are consistent with the fact that heterochromatic DNA breaks are preferentially repaired through homologous recombination [11, 36, 37] and CCAR2 involvement in this mechanism has already been reported [33].

Curiously, these events seem to be independent of SIRT1, even if this deacetylase was previoulsy reported to be involved in both conservative and non conservative DNA repair pathways [38, 39], but involve the Chk2 kinase activity towards KAP1. It's well established that CCAR2 interacts with the kinase Chk2 [7], which has among its targets also the transcriptional repressor KAP1 [18, 19]. In particular, Chk2 phosphorylates KAP1 on Ser473 decreasing the interaction between KAP1 and HP1 proteins: this post translational modification promotes HP1β mobilization and the reorganization of chromatin structure favoring the repair of DNA breaks inside heterochromatin [18, 19]. However, controversial data about the role of KAP1-S473 phosphorylation in the DNA damage response exist. Indeed other groups reported that this modification does not affect the binding of KAP1 with HP1 proteins [21, 40], but that it is involved in the maintenance of G2/M checkpoint upon IR [41]. Our data demonstrating the decrease of KAP1-HP1α interaction upon etoposide exposure in CCAR2+/+ cells and the induction of this association in CCAR2−/− cells, where the phosphorylation of S473 is reduced, further confirm the role of this modification in the regulation of KAP1-HP1 proteins association which finally impacts on chromatin structure leading to increased accessibility for repair factors. These data are in accordance with those previously reported [21] demonstrating that disruption of KAP1-HP1 association facilitates γH2AX foci resolution. In contrast we did not find any role of KAP1-S473 phosphorylation in G2/M checkpoint activation and sustainment, since both CCAR2+/+ and CCAR2−/− cells did not display significant differences in cell cycle checkpoint activation. However it is possible that the reduction of S473 observed in CCAR2−/− cells could not be sufficient to affect cell cycle checkpoints.

Curiously we found that, although the priming phosphorylation of Chk2 on T68 by ATM is not affected by CCAR2, the dimerization and the autophosphorylation on T387 of Chk2, essential for a full activity of Chk2 on its substrates [8], are reduced in CCAR2 ablated cells. This reduction of Chk2 activation finally leads to defective phosphorylation of KAP1 on S473 that can prevent chromatin relaxation and DNA repair. Of note, we found as previously reported [21] that KAP1 phosphorylated on S473 does not accumulate in DNA damage induced foci (data not shown), as expected because of its role in the induction of global chromatin relaxation upon DNA damage. Beside this, our results suggest that CCAR2 could be involved in the regulation of the interaction between Chk2 and its substrates. Indeed, in CCAR2-WT cells, Chk2-KAP1 association decreases in response to DNA damage, whereas it is induced in CCAR2 negative cells. This phenomenon could be explained with the fact that Chk2 releases its substrates after phosphorylation [8, 29, 30], but maintains the interactions in the case of defective catalytic activity.

As a consequence of these observations we hypothesize that CCAR2 could exerts a direct role in Chk2 activation, possibly favoring the proper conformational changes necessary for Chk2 dimerization and auto-phosphorylation; nevertheless, other proteins could be involved in this molecular mechanism, contributing to finely regulate Chk2 activities during the DNA damage response. Of note, CCAR2 is the first protein described to affect Chk2 dimerization without impairing the ATM activity on Chk2, even if our experiments revealed that ATM could play a role in regulating Chk2 activity through CCAR2. Indeed, in the analysis of Chk2 auto-phosphorylation, we found that overexpression of CCAR2 mutated in the ATM target site (CCAR2^T454A^ [2]) has a minor effect compared to CCAR2^WT^ overexpression; moreover, when we evaluated KAP1-phospho-S473 in U2OS-CCAR2 negative cells re-complemented with CCAR2^WT^ or CCAR2^T454A^ vectors, we found that CCAR2^T454A^ overexpression rescued the phosphorylation defect of CCAR2^−/−^ cells to a less extent than CCAR2^WT^ overexpression.

However, since we found that Chk2 pro-apoptotic activity is not affected by the presence of CCAR2, we do not know whether CCAR2 regulates in the same manner also Chk2 activity towards targets different from KAP1. Indeed it is possible that CCAR2 could be involved in the regulation of specific, but not all, Chk2 activities.

Collectively our data indicate that, in response to DNA damage, CCAR2 is required for the proper dimerization and activation of Chk2 which finally leads to Chk2-dependent KAP1 phosphorylation and heterochromatic DNA repair, possibly by the regulation of chromatin relaxation (Figure 6). These data illustrate a new mechanism of Chk2 activity regulation and further confirm the role of CCAR2 in the DDR, suggesting for this protein an important role in genomic stability maintenance, given that the majority of mutations and chromosomal aberrations of cancer cells reside in the heterochromatic regions of the genome [42]; for this, our studies may also support the controversial hypothesis that CCAR2 could act as a tumor suppressor gene [43].

## MATERIALS AND METHODS

### CCAR2−/− cells production by CRISPR/Cas9 system

To generate CCAR2−/− cell lines we used the CRISPR/Cas9 system [22]. For this, a 20nt sequence (5′-GGAGTGAGGTGGACCCGGTA-3′) complementary to exon 8 of genomic CCAR2 and verified by computational analyses to exclude OFF-target effects [44], was cloned into the gRNA_Cloning vector (Addgene plasmid 41824) according to the reported protocol [22]. The CCAR2-gRNA and human codon optimised Cas9 encoding vectors (Addgene 41815) were transfected in U2OS cells and 72h later analyzed by IF to determine the percentage of CCAR2-negative cells, and then subcloned. Clones were first screened by IF and WB and then the presence of *indel* was verified by sequencing. In this study we also used a BJ-hTERT clone knocked out for CCAR2 generated with the same system.

### Cell lines and treatments

Human osteosarcoma U2OS cells and U2OS AID-DIvA cells (a kind gift of Dr. G. Legube) were cultured as reported [7, 27]. BJ-hTERT human fibroblast cells were grown in DMEM/Medium199 (4:1) with 10% of fetal bovine serum and 10μg/ml Hygromycin B. The Chk2 inhibitor VRX0466617 was kindly provided by Dr Minmin Yang (Pharmablock) and added to cells at 100 μM 1h before treatments. Etoposide (TEVA) was used at 20 μM. FACS analyses were performed as described [26]. Irradiations were performed in an IBL437CO instrument equipped with a ^137^Ce source emitting a dose of 8 Gy/min.

### Expression vectors, siRNAs and tranfections

Vectors encoding CCAR2^WT^, CCAR2^T454A^, HA-Chk2 and FLAG-Chk2 were previously described [2, 31]. HP1α c-DNA was obtained from Addgene (plasmid 17652) and then cloned in the pcDNA3-FLAG vector. siRNAs against CCAR2 and SIRT1 were ON-TARGET plus SMART pool (Thermo Scientific Dharmacon), whereas those against HP1β were FlexiTube siRNA (Qiagen). Lipofectamine 2000 (Invitrogen) and Lipofectamine RNAiMAX (Invitrogen) were used for plasmids and siRNAs transfections, respectively, according to the manufacturer's instructions.

### Western blots, antibodies and immunoprecipitations

The NuPAGE system (Life Technologies) was used for western blot analyses and densitometric evaluations were performed with the ImageQuant 5.2 software (Molecular Dynamics). Quantification of protein levels were normalized to loading control and for phosphorylated proteins to total protein. Antibodies used in this study were: CCAR2 (Bethyl Laboratories or Cell Signaling Technology); phospho-Chk2-T68, phospho-Chk2-T387, Cleaved Caspase-9, KAP1, phospho-KAP1-S824, SIRT1, phospho-p53-S20 (Cell Signaling Technology); phospho-KAP1 S473 (Biolegend); 53BP1 (Novus), γH2AX and H3K9me3 (Upstate); FLAG (clone M2) and β-Actin (Sigma); HA (clone 12CA5, Roche); HP1β (Epigentek); phospho-ATM-S1981 (R&D); ATM (Epitomics); p53 (Santa Cruz, DO-7). Chk2 antibody (clone 44D4/21) was previously described [45] and used for IP. For western blot Chk2 antibody from MBL Intl Corp (DCS-270 and DCS-273) was used. IP experiments were carried out as described [46] except for the interaction between HP1α and KAP1 that was assayed after cell lysates sonication and co-immunoprecipitations of 53BP1 and H3K9me3 that were performed as reported [20].

### Immunofluorescence and γH2AX or 53BP1 foci enumeration

Cells grown on glass coverslips were fixed with paraformaldehyde, permeabilized with 0.2% Triton X-100, blocked in PBS, 5% BSA, 0.1% Tween 20, stained with anti γH2AX (Upstate) or anti-53BP1 antibodies (Novus Biologicals, 100-304) and counterstained with DAPI. For cyclin B1 staining cells were permeabilized with 0.5% Triton, blocked in 3% BSA and incubated with cyclin B1 (BD Pharmingen) and 53BP1 antibodies. Coverslips were scored by fluorescence microscopy and digital image acquisition on a Nikon Eclipse E1000 equipped with a DS-U3 CCD camera.

γH2AX and 53BP1 foci were stained by immunofluorescence in CCAR2+/+ and CCAR2−/− cells untreated or treated for 1h with etoposide and then released in drug free medium for the indicated time points. Foci were scored on >100 nuclei by fluorescence microscopy using a 100X magnification objective by two independent operators. Standard deviations were calculated on the mean values of at least three independent experiments. P values were determined by *t*-student test.

### G1/S and G2/M transition evaluation

To evaluate G1/S transition, DNA replicating cells were detected with the Click-iT EdU assay kit (Life Technologies). Cells were treated with etoposide for 1h, released in EdU containing medium for 4h and stained according to manufacturer's instruction. For G2/M transition, etoposide treated cells were released in medium containing 100ng/ml of nocodazole to trap checkpoint defective cells. Mitotic cells were stained with an Alexa Fluor-488 conjugated anti phospho-histone-H3 (S10) antibody (Cell Signaling).

## SUPPLEEMENTARY MATERIAL FIGURES


