# Supplementary material for: CCAR2/DBC1 is required for Chk2-dependent KAP1 phosphorylation and repair of DNA damage
Source: Oncotarget. 2015 Jun 10;6(19):17817–31. doi: 10.18632/oncotarget.4417 (PMC4627348; doi:10.18632/oncotarget.4417)
Supplement: Supplementary file 1 [file oncotarget-06-17817-s001.pdf]

CCAR2/DBC1 is required for Chk2-dependent KAP1 phosphorylation and repair of DNA damage

Supplementary Material

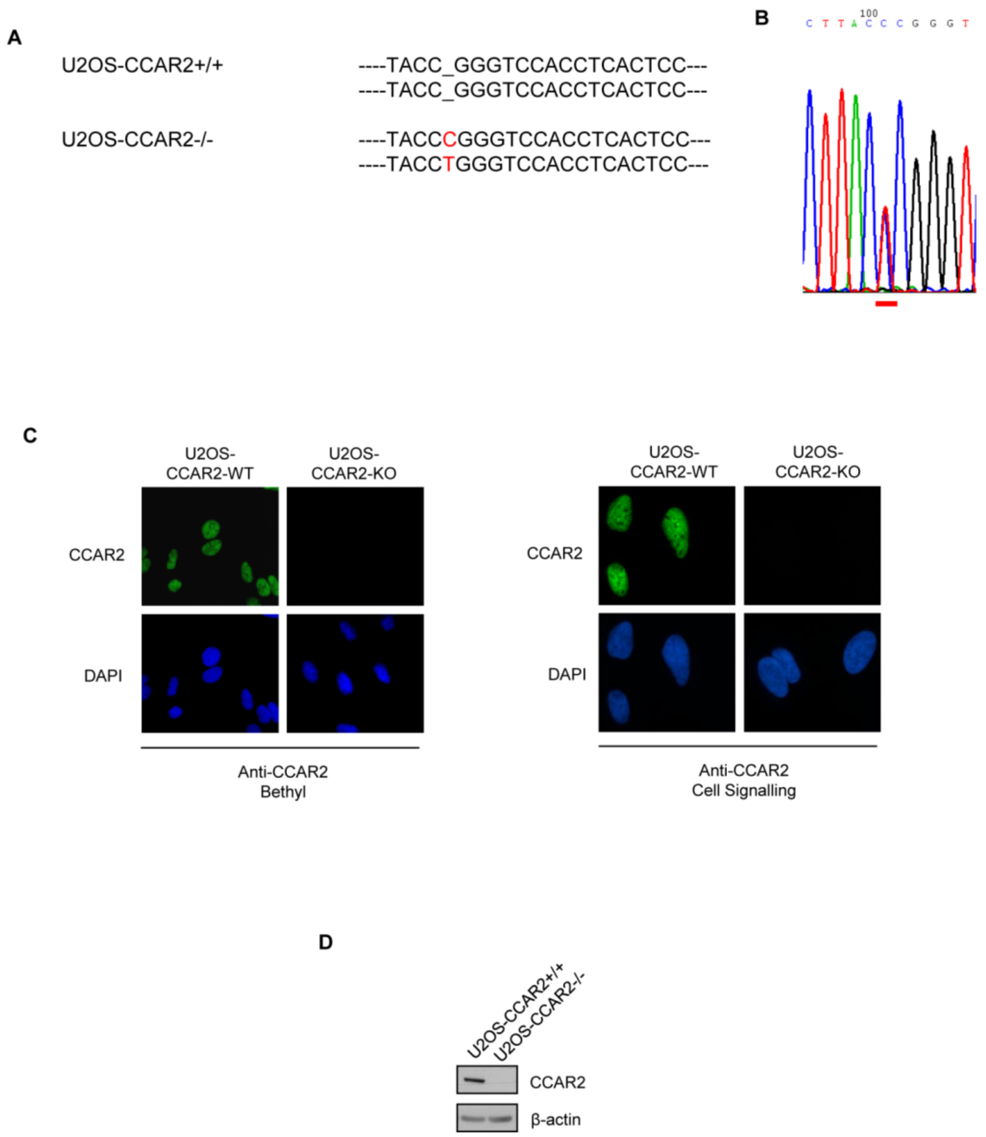

Supplementary Figure 1. Validation of U2OS-CCAR2-/- cells.

U2OS cells knock-out for CCAR2 (U2OS-CCAR2-/-) were prepared using the CRISPR/Cas9 system. Sequence alignment of the clone used for our studies is shown in **A,** whereas sequence chromatogram is reported in **B**. Loss of CCAR2 protein was demonstrated by immunofluorescence (**C**) performed with two different antibodies, one recognizing an epitope at the N- terminus (right) and the other developed against a C-terminal sequence (left), and by western blot analyses (**D**).

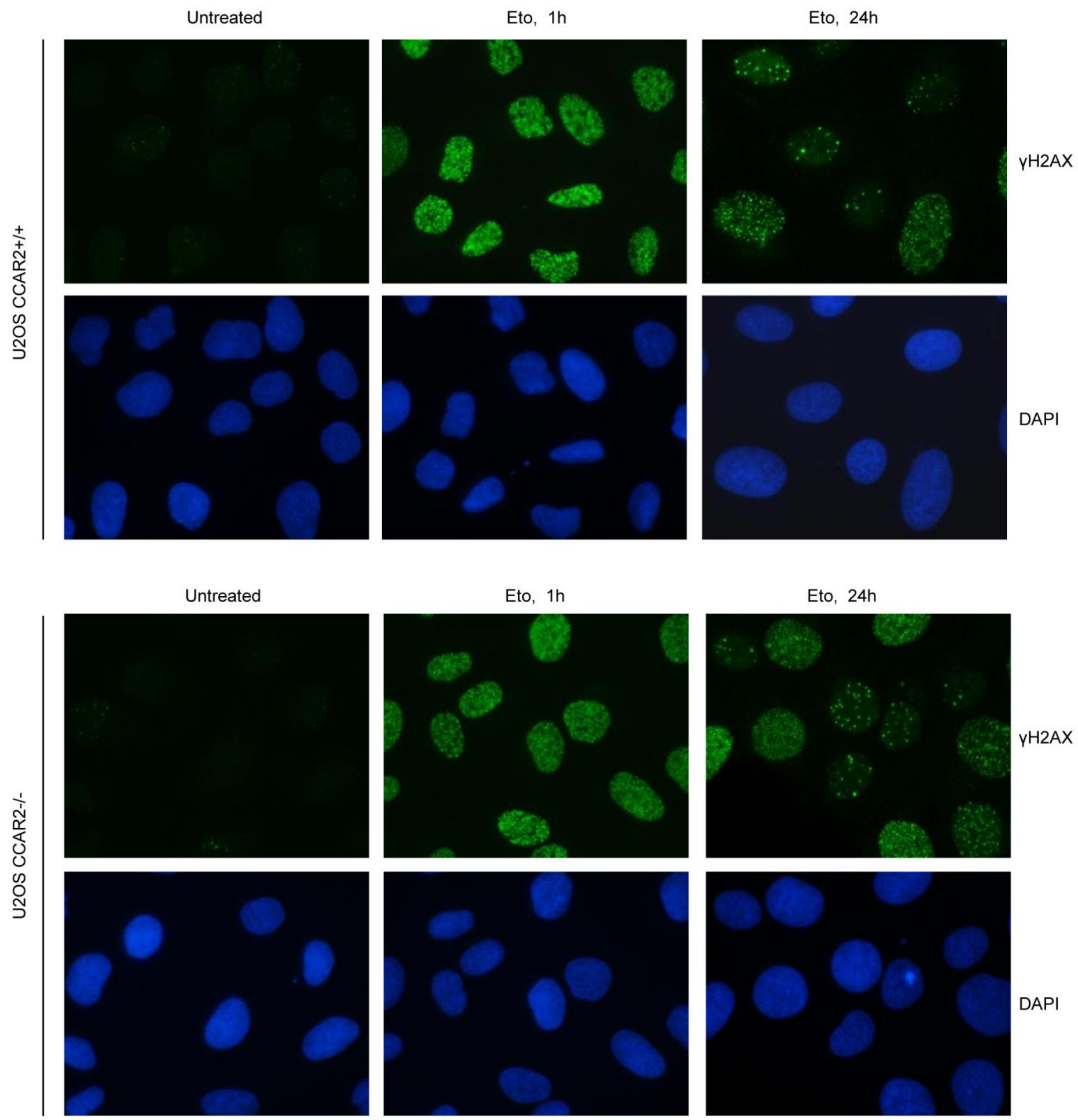

Supplementary Figure 2. Analysis of γH2AX foci in response to etoposide treatment.
U2OS-CCAR2+/+ and U2OS-CCAR2-/- cells were treated with etoposide for 1h and released in drug-free medium for 24h; γH2AX foci were analyzed by immunofluorescence.

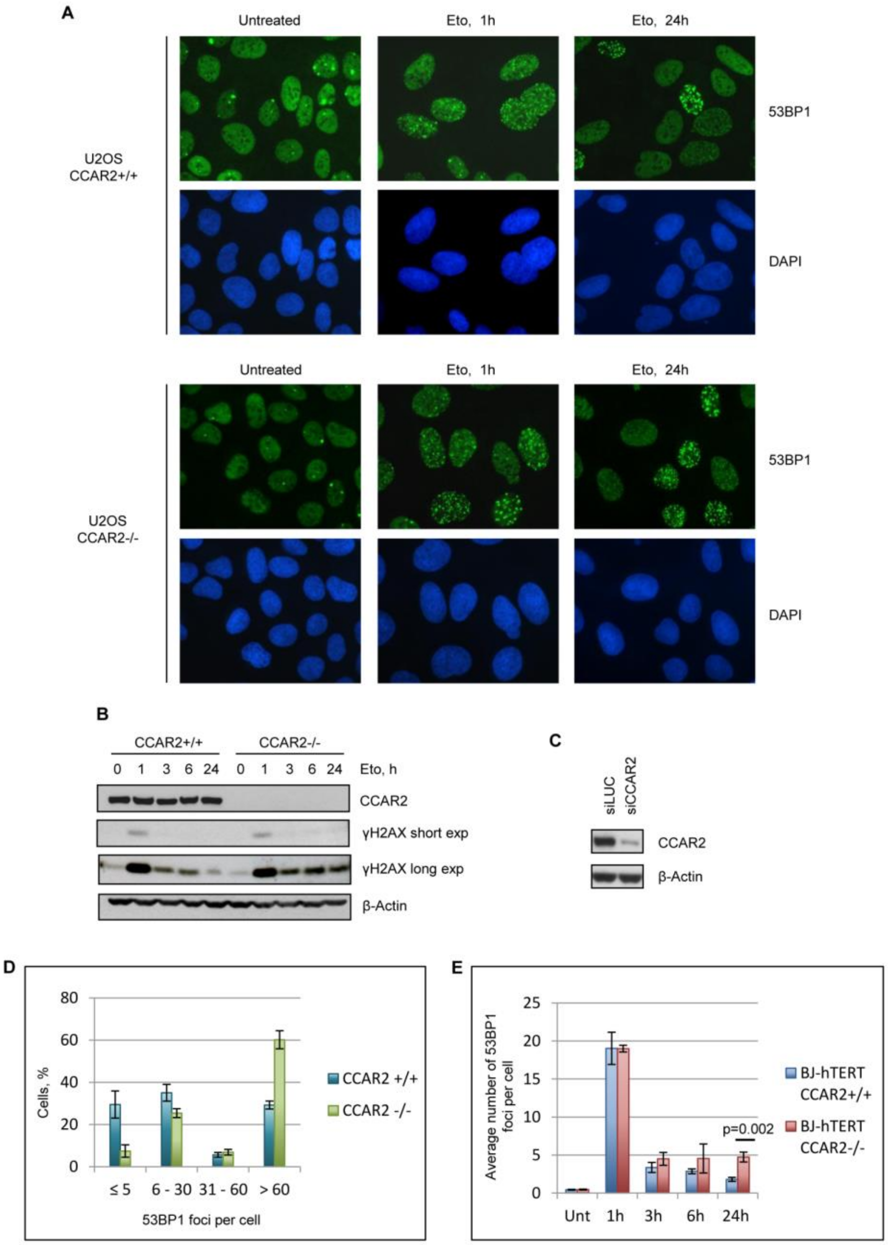

Supplementary Figure 3. **U2OS and BJ-hTERT CCAR2-/- cells show accumulation of 53BP1 foci and γH2AX levels.**

U2OS-CCAR2+/+ and U2OS-CCAR2-/- cells were exposed for 1h to 20µM etoposide, washed and incubated in drug-free medium for the indicated time points. 53BP1 foci were analyzed by immunofluorescence (**A**) and γH2AX levels by western blot (**B**). Western blot showing CCAR2 depletion in U2OS cells (**C**). Chart depicting 53BP1 foci distribution in the population of U2OS CCAR2+/+ and CCAR2-/- cells treated with etoposide for 1h and then released in drug-free medium for 24h (**D**). Time course analysis of 53BP1 foci in BJ-hTERT CCAR2+/+ and CCAR2-/- cells exposed to etoposide for 1h and then released in drug-free medium for 3, 6 and 24h.

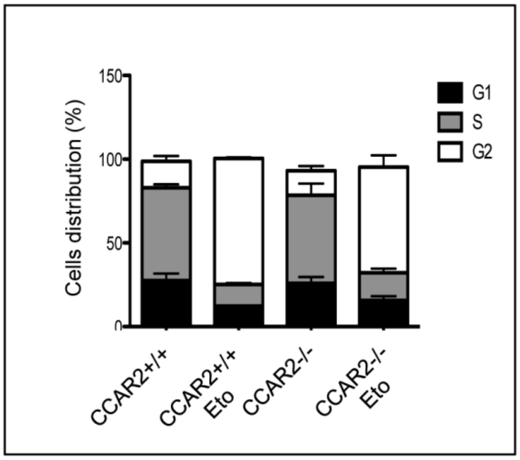

Supplementary Figure 4. **U2OS CCAR2+/+ and CCAR2-/- show similar cell cycle profiles.**

FACS analyses of U2OS-CCAR2+/+ and CCAR2-/- untreated or upon 1h etoposide treatment and release in drug-free medium for 24h.

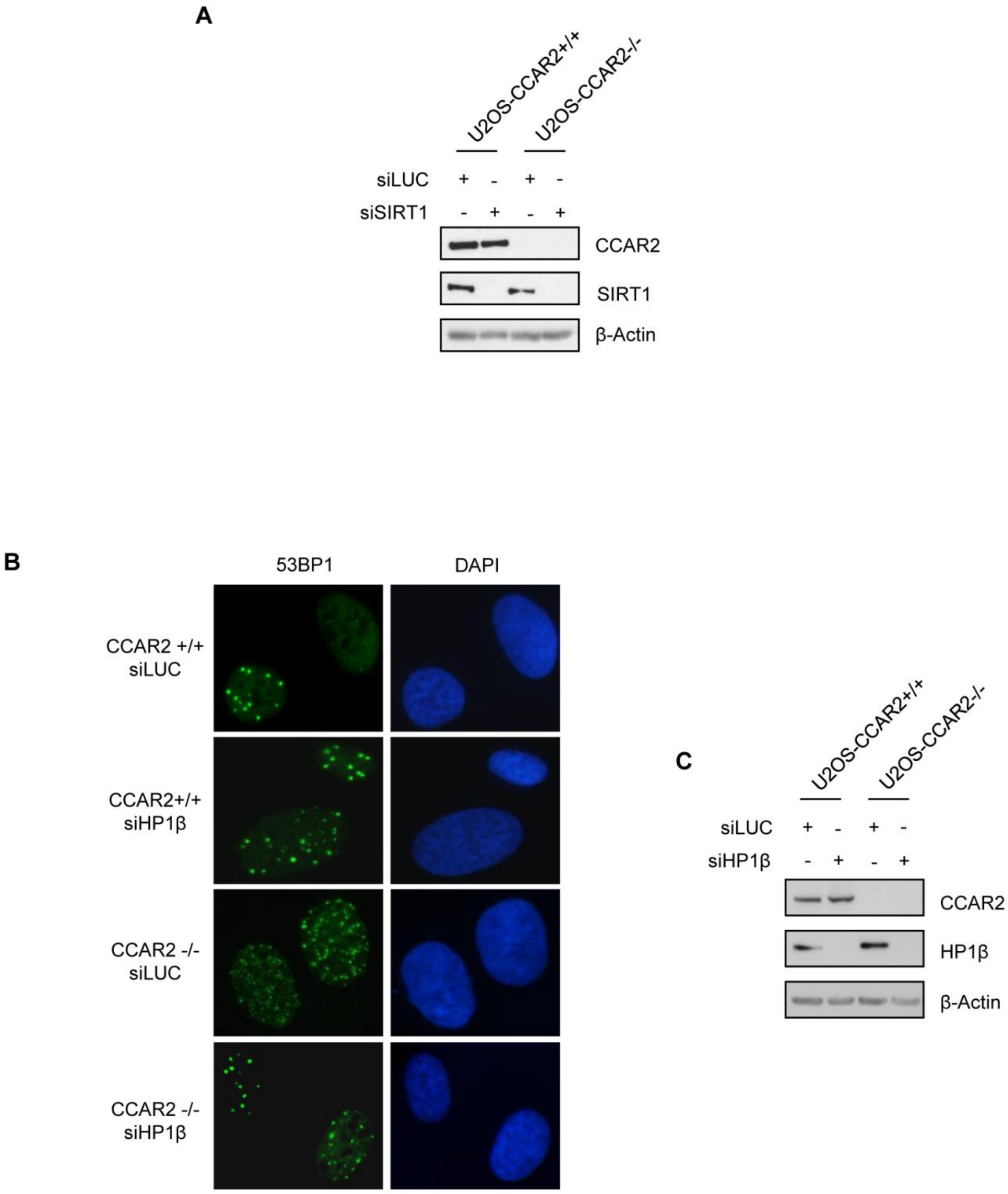

Supplementary Figure 5. SIRT1 is not involved in CCAR2-mediated DNA repair.

(**A**) Western blot analysis to detect SIRT1 depletion in CCAR2+/+ and CCAR2-/- cells. (**B**) Example of 53BP1 staining of U2OS-CCAR2+/+ and CCAR2-/- cells transfected with control or HP1β siRNA. (**C**) Western blot to detect HP1β depletion.

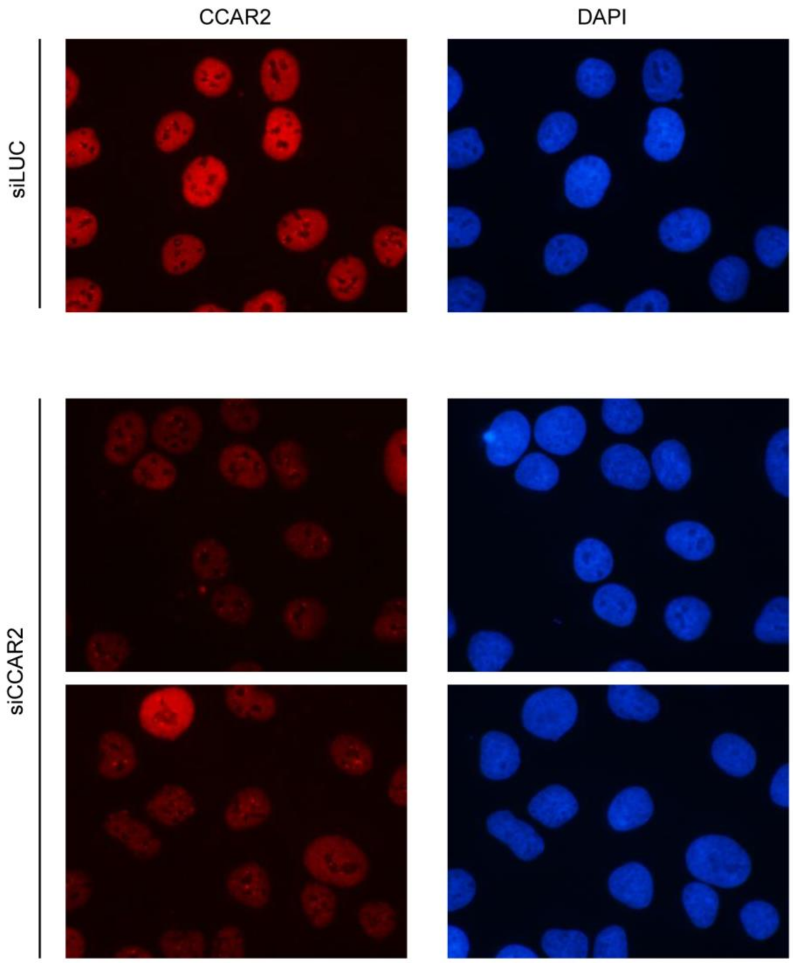

Supplementary Figure 6. CCAR2 does not affect DNA repair in AID-DiVA cells.
Immunofluorescence staining of AID-DiVA cells transfected with control or CCAR2 siRNA.

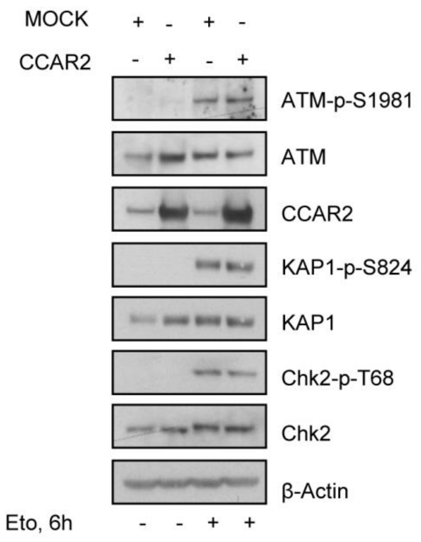

Supplementary Figure 7. CCAR2 does not affect ATM activity.

U2OS cells transfected with mock or CCAR2 encoding vectors and treated or not with etoposide were analyzed by WB with the indicated antibodies.

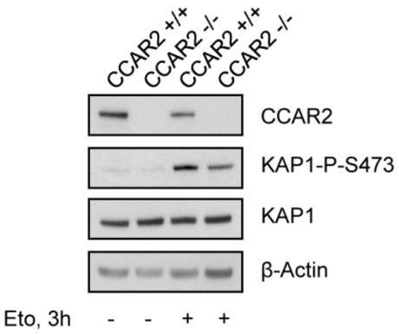

Supplementary Figure 8. CCAR2 absence reduces KAP1 phosphorylation on S473.

BJ-hTERT-CCAR2+/+ and CCAR2-/- cells were treated with etoposide for 3h and the phosphorylation of KAP1 on S473 was analyzed by western blot.

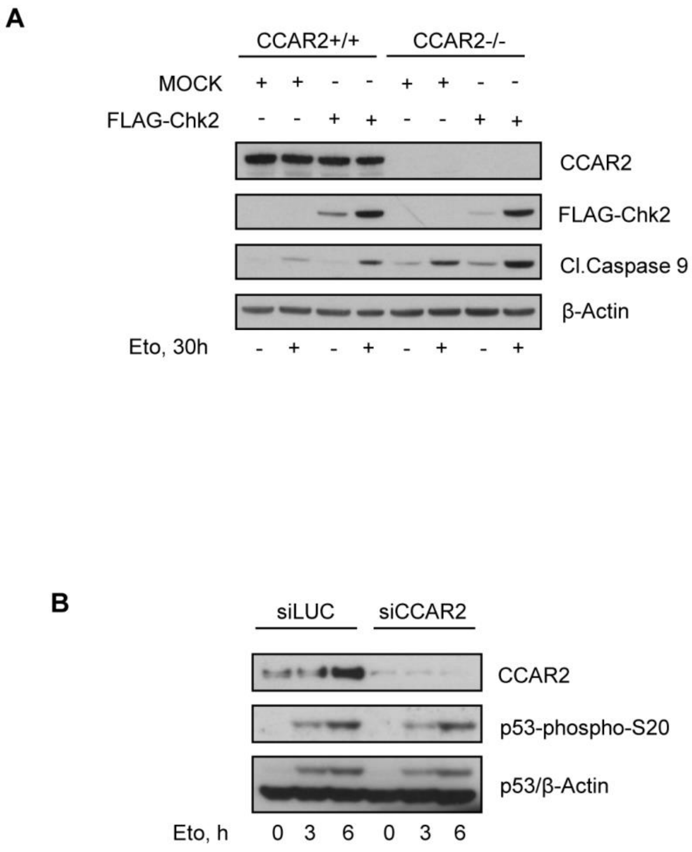

Supplementary Figure 9. Chk2-dependent apoptosis and p53 phosphorylation in CCAR2+/+ and CCAR2-/- cells.

(**A**) Chk2 protein was overexpressed in CCAR2+/+ and CCAR2-/- cells and apoptosis was analyzed in response to etoposide treatment by evaluation of caspase 9 cleavage. (**B**) p53-S20 phosphorylation was analyzed in U2OS cells transfected with control or CCAR2 siRNA and exposed to etoposide for the indicated time points.

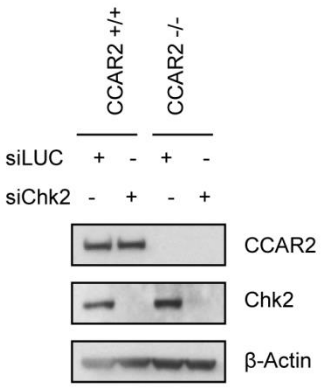

Supplementary Figure 10. Chk2 depletion affects DNA damage repair.

Western blot analyses of U2OS-CCAR2+/+ and CCAR2-/- cells transfected with control or Chk2 siRNAs.
